# Using Sequence Variants in Linkage Disequilibrium with Causative Mutations to Improve Across-Breed Prediction in Dairy Cattle: A Simulation Study

**DOI:** 10.1534/g3.116.027730

**Published:** 2016-06-13

**Authors:** Irene van den Berg, Didier Boichard, Bernt Guldbrandtsen, Mogens S. Lund

**Affiliations:** * Department of Molecular Biology and Genetics, Center for Quantitative Genetics and Genomics, Aarhus University, DK-8830 Tjele, Denmark; † Génétique Animale et Biologie Intégrative (GABI), French National Institute for Agricultural Research (INRA), AgroParisTech, Université Paris Saclay, 78350 Jouy-en-Josas, France

**Keywords:** across-breed prediction, sequence data, genomic relationships, linkage disequilibrium, genomic selection, GenPred, shared data resource

## Abstract

Sequence data are expected to increase the reliability of genomic prediction by containing causative mutations directly, especially in cases where low linkage disequilibrium between markers and causative mutations limits prediction reliability, such as across-breed prediction in dairy cattle. In practice, the causative mutations are unknown, and prediction with only variants in perfect linkage disequilibrium with the causative mutations is not realistic, leading to a reduced reliability compared to knowing the causative variants. Our objective was to use sequence data to investigate the potential benefits of sequence data for the prediction of genomic relationships, and consequently reliability of genomic breeding values. We used sequence data from five dairy cattle breeds, and a larger number of imputed sequences for two of the five breeds. We focused on the influence of linkage disequilibrium between markers and causative mutations, and assumed that a fraction of the causative mutations was shared across breeds and had the same effect across breeds. By comparing the loss in reliability of different scenarios, varying the distance between markers and causative mutations, using either all genome wide markers from commercial SNP chips, or only the markers closest to the causative mutations, we demonstrate the importance of using only variants very close to the causative mutations, especially for across-breed prediction. Rare variants improved prediction only if they were very close to rare causative mutations, and all causative mutations were rare. Our results show that sequence data can potentially improve genomic prediction, but careful selection of markers is essential.

For accurate genomic prediction, it is important to have a large reference population (Goddard and Hayes 2009). Therefore, combining reference populations of several breeds could improve across-breed prediction. The level of linkage disequilibrium (LD) across breeds is, however, substantially lower than within breed. Therefore, with the current 50K and high-density (HD) single nucleotide polymorphism (SNP) chips, combining reference populations results in increased prediction accuracy only for closely related breeds, such as different Nordic Red populations (Brøndum *et al.* 2011), while, for more distant breeds, such as Holstein and Jersey, the accuracy of across-breed prediction is low (Hayes *et al.* 2009; Erbe *et al.* 2012; Lund *et al.* 2014). Increasing the SNP density from 50K to HD was expected to increase prediction accuracy across breeds, because, at the marker density of the HD chip, LD between causative mutations and markers should be sufficient for across-breed prediction (de Roos *et al.* 2008). In practice, however, only small or no differences between prediction accuracies using the 50K or HD were found (Erbe *et al.* 2012; Hozé *et al.* 2014). By containing causative mutations as well as variants in very high LD with the causative mutations, sequence data could potentially make accurate across-breed prediction feasible. The increasing number of whole-genome sequences available will enable the use of sequence data in breeding programs. With the 1000 Bull Genomes Project (Daetwyler *et al.* 2014), a large reference population of sequences is available for imputation of SNP chip genotypes to whole-genome sequence, which can subsequently be used for genome-wide association studies and genomic prediction.

While most variants in the sequence have a low minor allele frequency (MAF), the variants on the SNP chips are selected to have high MAF (Matukumalli *et al.* 2009). Differences in MAF between markers and causative mutations would limit the maximum LD achievable between markers and causative mutations. The abundance of low MAF variants in the sequence data could suggest that at least some of the causative mutations have low MAF. Using sequence data to predict these variants could therefore increase prediction accuracy, either by inclusion of the causative variants, or by achieving a stronger LD with low-MAF sequence variants near the causative mutations and the causative mutations themselves. Druet *et al.* (2014) found that using sequence data for genomic prediction leads to a higher prediction accuracy for rare causative mutations.

Sequence data does not, however, necessarily contain all causative variants for various reasons, including filtering and imputation errors. Furthermore, even if all causative variants were present, and even if a trait is very polygenic, the number of variants directly affecting that trait is only a very small proportion of all variants present in the sequence. Therefore, the majority of sequence variants used for genomic prediction will be, at most, in imperfect LD with the causative mutations. As a consequence, the prediction reliability will be lower than the prediction reliability if only causative mutations were used. The reduction in prediction reliability as a consequence of imperfect LD between causative mutations and markers can be quantified by the regression of genomic relationships at markers used for prediction on genomic relationships at causative mutations (de los Campos *et al.* 2013). We used this regression to investigate the potential benefits of using sequence data for within- and across-breed prediction in five French and Danish dairy cattle breeds.

In this paper, we focus on the influence on across-breed prediction of LD between causative mutations and markers. While LD is an important factor, the accuracy of across-breed prediction is also influenced by other factors. Only part of the causative mutations segregating within breed are segregating across breeds (Raven *et al.* 2014), and causative mutations that do segregate across breeds can have different effects in different breeds, due to, *e.g.*, epistasis and dominance. Therefore, results in this paper should be interpreted as maximum gains that could be achieved by using sequence variants near the causative mutations.

The objective of this study was to use actual sequence data from representative individuals of different breeds to investigate the potential benefits of sequence data for prediction of genomic relationships compared to the use of 50K and HD data, focusing on the loss in prediction reliability due to the use of markers in imperfect LD with the causative mutations. Because the advantages of using sequence data are expected to be larger in populations with low LD than in populations with high LD, within- and across-breed predictions were compared in several scenarios. We simulated several scenarios, varying the number of causative mutations, and the MAF of the causative mutations. Different sets of markers were subsequently used, with sequence variants on varying distances from the causative mutations, varying the MAF of the markers, or variants from the 50K and HD SNP chips. First, we validated the formula proposed by de los Campos *et al.* (2013) to estimate the loss in prediction reliability for across-breed prediction. Subsequently, we applied this formula to a wider range of scenarios and breeds.

## Materials and Methods

Two datasets were used for different parts of the study. Both datasets consisted of sequence data on chromosome 1. The first set (SEQ, Supplemental Material, File S1) contained nonimputed sequence data of 122 Holstein (HOL), 27 Jersey (JER), 28 Montbéliarde (MON), 23 Normande (NOR), and 45 Danish Red (RDC) bulls. These bulls were the first bulls to be sequenced in France and Denmark, and were major ancestors contributing to each breed. While all JER and RDC bulls were Danish bulls, and all MON and NOR were French, HOL bulls originated from different populations. There were 41 French, 22 Danish, 22 American, eight Canadian, eight German, three Italian, two Swedish, and one Finnish HOL bulls. Variant calling was done using GATK (DePristo *et al.* 2011). There were 1,475,541 biallelic SNP and indels on chromosome (BTA) 1 segregating in at least one of the five breeds. Causative mutations were simulated by randomly sampling 100 or 250 variants, either out of all variants (C_100_ and C_250_) or out of all variants with a MAF below 0.10 (C_100LM_ and C_250LM_).

The second set (IMP, File S2) consisted of imputed sequences of 1230 JER and 961 RDC bulls. Imputation was done in two steps, using IMPUTE2 (Howie *et al.* 2012), first from 50K to HD, and subsequently to full sequence. More details on the imputation can be found in Höglund *et al.* (2014). After filtering out variants with an IMPUTE2 INFO score below 0.9, there were 1,150,905 variants segregating on chromosome 1. The majority of these variants were segregating in both JER and RDC, while this was not the case for the variants in the SEQ data. To make the dataset comparable, only a subset of the data were used for analyses, containing 247,141 variants for which the percentages of variants segregating in both JER and RDC, in JER but not in RDC, in RDC but not in JER, and neither in JER nor in RDC, were equal to those in SEQ. For the individuals in IMP, phenotypes were simulated as following. First, 50, 100, or 250 variants were randomly selected from all 247,141 variants as causative mutations for scenarios C_50_, C_100_, and C_250_, respectively. The effects were then drawn from a normal distribution ∼N(0,1). Subsequently, true breeding values (TBV) were obtained by summing the effects of the causative mutations, and a random error was added to the TBV to obtain a phenotype, so that the heritability = 0.8.

**Figure 1:**
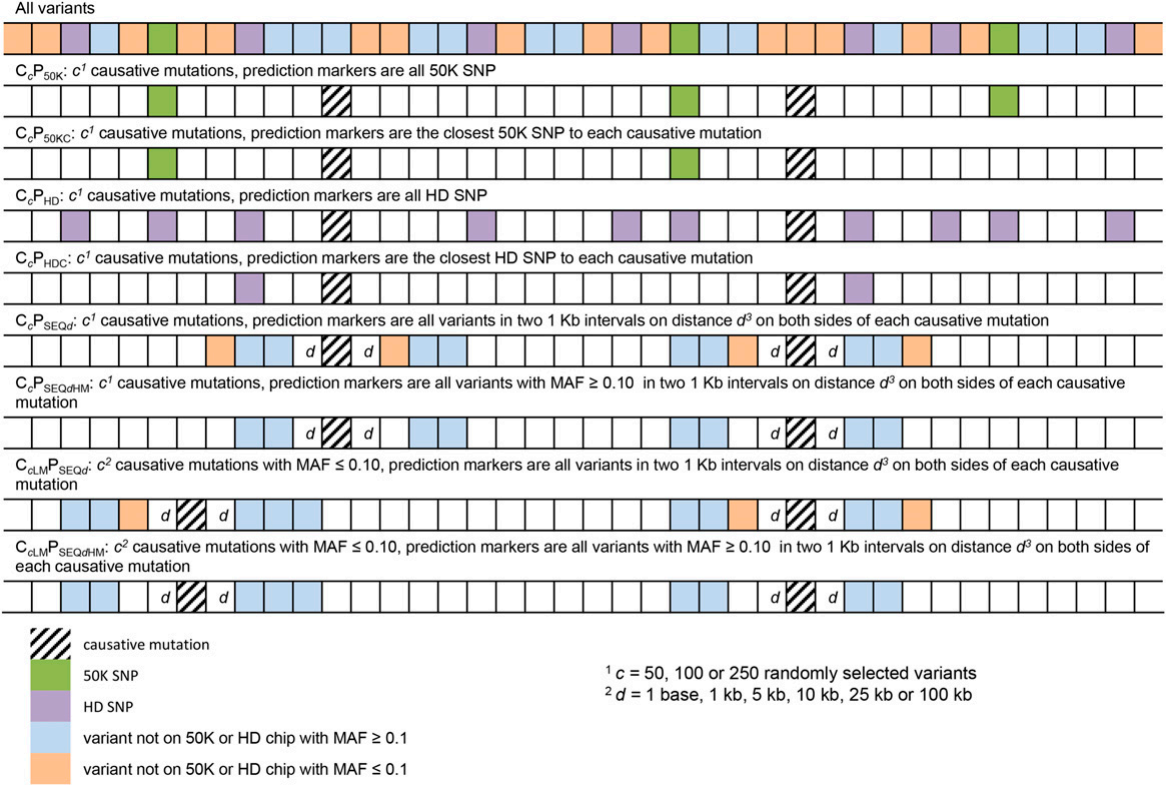
Description of the different scenarios. Scenarios varied according to the number (*c*) and minor allele frequency (MAF) of the causative mutations, and to the nature (from sequence or from chip), MAF, and distance to causative mutations of prediction variants.

For the SEQ dataset, markers were selected according to one of the following scenarios. In scenarios P_50K_ and P_HD_, markers were either all variants on BTA1 present on the Illumina BovineSNP50 Beadchip, or on the Illumina BovineHD Beadchip. In scenarios P_50KC_ and P_HDC_, only the closest variant of each causative mutation present on the 50K or the HD SNP chip were selected. In the remaining 16 scenarios, selected markers were all markers present in two 1-kb intervals on both sides of each causative mutation. The distance between the beginning of the intervals and the causative mutations (*d*) was 1 base, 1 kb, 5 kb, 10 kb, 25 kb, or 100 kb, and the intervals contained either all variants (P_SEQ_*_d_*), or only the variants with a MAF ≥ 0.10 (P_SEQ_*_d_*_HM_) present in the intervals. Each of these scenarios was repeated 50 times. For the IMP scenarios, the analysis was restricted to the P_50K_, P_HD_, P_50KC_, P_HDC_, P_SEQ1kb_, P_SEQ10kb_, and P_SEQ25kb_ scenarios, with 20 repeats per scenario. An overview of the subsets of variants used from which causal loci and markers were selected is given in Table 1. Figure 1 gives an overview of all different scenarios.

**Table 1:** Subsets of variants

Variants	*nVariants*	Overall	HOL	JER	MON	NOR	RDC
SEQ	1,475,541	0.12	0.11	0.10	0.11	0.11	0.12
IMP	247,141	0.10	—	0.09	—	—	0.10
50K	2863	0.26	0.25	0.20	0.23	0.24	0.25
HD	44,540	0.27	0.25	0.20	0.23	0.24	0.25
MAF ≤ 0.1	907,484	0.03	0.02	0.04	0.03	0.03	0.03
MAF ≥ 0.1	568,057	0.27	0.26	0.21	0.23	0.23	0.26

Number of variants (*nVariants*) and corresponding average minor allele frequencies (MAF) according to variant classes. HOL, Holstein; JER, Jersey; MON, Montbéliarde; NOR, Normande; RDC, Danish Red.

For each scenario, two genomic relationship matrices (**G**) were constructed, using either the causal loci or the markers following VanRaden (2008):

$$G=\frac{ZZ’}{2\sum p_{j}(1-p_{j})},$$

where **G** is the genomic relationship matrix at causative mutations or markers, **Z** is a standardized genotype matrix with the causative mutations or markers, and *p_j_* the allele frequency of the alternative allele for locus *j*. For individual *i* and locus *j*, $z_{ij}=n_{ij}-2p_{j}$, with *n_ij_* equal to the number of alternative alleles (0, 1, or 2). Allele frequencies were computed from genotypes of all individuals used to construct the genomic relationship matrix.

The genomic relationship matrices were constructed for all individuals in each breed, and for each pairwise combination of breeds. Only variants segregating in at least one of the breeds were used to construct the genomic relationship matrices. Subsequently, the loss in prediction reliability resulting from the use of markers rather than the causal loci was quantified following de los Campos *et al.* (2013):

$${\bar{R}}_{}^{2}\leq R_{}^{2}*RF,$$

where the difference in reliability between prediction using markers in imperfect LD with the causative mutations (${\bar{R}}_{}^{2}$), and the prediction (*R^2^*) if markers were in perfect LD with the causative mutations, is quantified by a reliability factor (RF). RF was either obtained (RF_O_) by estimating ${\bar{R}}_{}^{2}$ and *R^2^*, or predicted as RF_P_ = $1-{\left(1-b\right)}^{2}$. The *b* in the RF_P_ is the regression coefficient of the genomic relationships at markers on the genomic relationship markers at the causative mutations:

$${\bar{G}}_{n+1,i}=b_{n+1}G_{n+1,i}+{\text{ξ}}_{n+1,i},\;\left(i=1,\ldots ,\;n\right),$$

where ${\bar{G}}_{n+1,i}$ and $G_{n+1,i}$ are the genomic relationships between individual *n*+1 and individuals 1 to *n* at the markers and the causative mutations, respectively, and $\xi_{n+1,i}$ a random residual. First, *b* was computed for each individual. Subsequently, RF_P_ was computed within replicate, using the *b* averaged across individuals. Finally, RF_P_ was averaged across replicates.

To estimate RF_O_, the IMP dataset was split into training and validation sets, using either JER to predict genomically estimated breeding values (GEBVs) of RDC, or prediction of JER GEBVs using phenotypic information of RDC. GEBVs and variance components were estimated using Average Information Restricted Maximum Likelihood (AI-REML) (Jensen *et al.* 1997) as implemented in DMU (Madsen and Jensen 2013):

y=1μ+Zg+e,

where μ was the overall mean, **Z** an incidence matrix linking phenotypes with the vector that contains the additive genetic effects $g∼N(0,G*{\text{σ}}_{g}^{2})$, and **e** a vector with random residuals $e∼N\text{(}0,{\text{σ}}_{e}^{2}\text{)}$. The reliability was estimated as the squared correlation between GEBVs and TBVs. The genomic heritability was computed as $\frac{{\text{σ}}_{g}^{2}}{{\text{σ}}_{g}^{2}+{\text{σ}}_{e}^{2}}$.

### Data availability

The authors state that all data necessary for confirming the conclusions presented in the article are represented fully within the article.

## Results

### Genomic relationships within and across breeds

**Figure 2:**
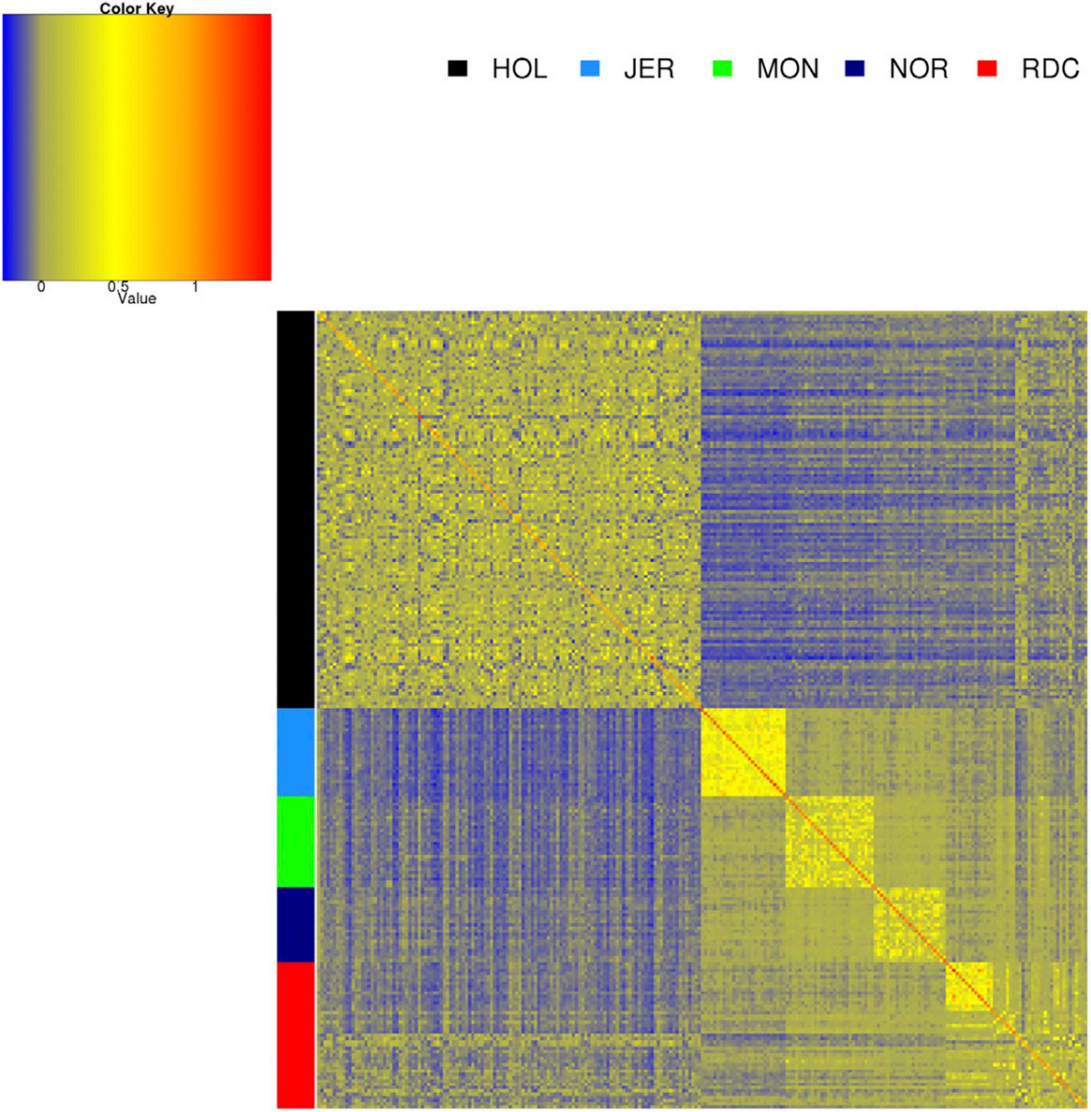
Genomic relationships within and across breed, using HD markers. HOL, Holstein; JER, Jersey; MON, Montbéliarde; NOR, Normande; RDC, Danish Red.

Figure 2 shows the genomic relationships using HD markers within and across breeds. HOL bulls originated from several countries, and within breed relationships were smaller than in the other breeds. Genomic relationships between other breeds were larger than with HOL, except for some of the RDC bulls. Within RDC, some of the individuals were highly related, while others were less related, which confirms the admixed nature of the breed.

### Variants segregating across breeds

The percentage of simulated causative mutations that were segregating in each breed, and shared between each combination of two breeds, is shown in Table 2. The largest number of causative mutations segregated within HOL and RDC, 78% and 77%, respectively, when causative mutations were selected out of all variants, and 64% and 62% when causative mutations were selected out of all variants with a MAF below 0.10. The smallest percentages were observed in JER, with 54% and 32% without and with restriction on MAF, respectively. HOL and RDC shared the largest number of causative mutations, and, for each breed, the percentage of shared causative mutations was highest with HOL and RDC and lowest with JER.

**Table 2:** Sharing of causative mutations between breeds

	All Variants					MAF ≤ 0.1				
Breed	HOL	JER	MON	NOR	RDC	HOL	JER	MON	NOR	RDC
HOL	78					64				
JER	48	54				23	32			
MON	52	41	60			27	16	41		
NOR	52	41	45	60		26	16	19	39	
RDC	64	48	52	52	77	43	23	29	27	62

Percentage of simulated causative mutations that was segregating in each breed (diagonal) and shared between breeds (below diagonal) according to minor allele frequency (MAF). HOL, Holstein; JER, Jersey; MON, Montbéliarde; NOR, Normande; RDC, Danish Red.

### Reliabilities for different sets of markers

Table 3 shows the reliabilities, heritabilities, and RF estimated by cross-validation, and compares the observed RF (RF_O_) with the RF predicted by the formula (RF_P_). Reliabilities were very high for the causative mutations, ranging from 0.73 for prediction of RDC with a JER reference population with 250 causative mutations, to 0.95 for prediction of JER with a RDC reference population with 50 causative mutations. Reliabilities were substantially lower for all sets of markers. The highest reliabilities using markers were obtained with P_SEQ1kb_, and decreased when the distance between markers and causative mutations increased. Reliabilities equaled 0.38, 0.23, and 0.09 for P_SEQ1kb_, P_SEQ10kb_, and P_SEQ25kb_, respectively, when averaged across breeds and number of causative mutations. Reliabilities were lowest for P_50K_ (0.06) and P_HD_ (0.10), averaged across breeds and number of causative mutations. Using only the markers closest to the causative mutations rather than all 50K or HD markers increased reliability, especially for P_HDC_, with reliabilities of 0.11 and 0.30 for P_50KC_ and P_HDC_, respectively.

**Table 3:** Reliabilities and observed and prediction reliability factors for different sets of markers

ncaus	Train	Val	Set	Reliability	Heritability	RF_O_	RF_P_
50	JER	RDC	C_c_	0.85 (0.02)	0.79 (0.01)	—	—
			P_SEQ1kb_	0.38 (0.03)	0.75 (0.01)	0.45 (0.04)	0.39 (0.02)
			P_SEQ10kb_	0.27 (0.03)	0.72 (0.01)	0.31 (0.04)	0.25 (0.02)
			P_SEQ25kb_	0.13 (0.02)	0.71 (0.01)	0.15 (0.03)	0.16 (0.01)
			P_50K_	0.04 (0.01)	0.78 (0.01)	0.05 (0.02)	0.03 (0.00)
			P_50KC_	0.09 (0.02)	0.54 (0.02)	0.11 (0.02)	0.20 (0.01)
			P_HD_	0.08 (0.02)	0.78 (0.01)	0.10 (0.03)	0.03 (0.00)
			P_HDC_	0.31 (0.04)	0.61 (0.02)	0.36 (0.04)	0.34 (0.02)
	RDC	JER	C_c_	0.95 (0.01)	0.78 (0.01)	—	—
			P_SEQ1kb_	0.47 (0.04)	0.70 (0.01)	0.50 (0.04)	0.38 (0.02)
			P_SEQ10kb_	0.26 (0.03)	0.66 (0.02)	0.28 (0.04)	0.23 (0.02)
			P_SEQ25kb_	0.13 (0.03)	0.61 (0.02)	0.14 (0.03)	0.14 (0.01)
			P_50K_	0.09 (0.02)	0.75 (0.01)	0.10 (0.02)	0.02 (0.00)
			P_50KC_	0.14 (0.03)	0.39 (0.02)	0.15 (0.03)	0.18 (0.01)
			P_HD_	0.14 (0.03)	0.77 (0.01)	0.15 (0.03)	0.02 (0.00)
			P_HDC_	0.40 (0.04)	0.51 (0.03)	0.42 (0.04)	0.33 (0.02)
100	JER	RDC	C_c_	0.84 (0.01)	0.79 (0.01)	—	—
			P_SEQ1kb_	0.36 (0.03)	0.79 (0.01)	0.43 (0.03)	0.43 (0.02)
			P_SEQ10kb_	0.24 (0.03)	0.79 (0.01)	0.29 (0.03)	0.26 (0.01)
			P_SEQ25kb_	0.08 (0.01)	0.78 (0.01)	0.09 (0.01)	0.17 (0.01)
			P_50K_	0.06 (0.01)	0.79 (0.01)	0.07 (0.01)	0.05 (0.00)
			P_50KC_	0.09 (0.02)	0.61 (0.01)	0.11 (0.02)	0.21 (0.01)
			P_HD_	0.11 (0.02)	0.79 (0.01)	0.13 (0.02)	0.06 (0.00)
			P_HDC_	0.28 (0.03)	0.68 (0.01)	0.34 (0.03)	0.37 (0.01)
	RDC	JER	C_c_	0.92 (0.01)	0.79 (0.01)	—	—
			P_SEQ1kb_	0.47 (0.04)	0.75 (0.01)	0.50 (0.04)	0.41 (0.02)
			P_SEQ10kb_	0.32 (0.03)	0.72 (0.01)	0.35 (0.03)	0.24 (0.01)
			P_SEQ25kb_	0.12 (0.02)	0.68 (0.01)	0.13 (0.02)	0.14 (0.01)
			P_50K_	0.07 (0.01)	0.76 (0.01)	0.08 (0.01)	0.03 (0.00)
			P_50KC_	0.14 (0.02)	0.47 (0.01)	0.15 (0.02)	0.19 (0.01)
			P_HD_	0.14 (0.02)	0.78 (0.01)	0.15 (0.02)	0.03 (0.00)
			P_HDC_	0.37 (0.04)	0.59 (0.01)	0.40 (0.04)	0.36 (0.01)
250	JER	RDC	C_c_	0.73 (0.02)	0.79 (0.01)	—	—
			P_SEQ1kb_	0.26 (0.02)	0.81 (0.01)	0.35 (0.03)	0.47 (0.01)
			P_SEQ10kb_	0.12 (0.01)	0.80 (0.01)	0.17 (0.02)	0.32 (0.01)
			P_SEQ25kb_	0.04 (0.01)	0.81 (0.01)	0.06 (0.01)	0.23 (0.01)
			P_50K_	0.04 (0.01)	0.79 (0.01)	0.05 (0.01)	0.13 (0.00)
			P_50KC_	0.07 (0.01)	0.74 (0.01)	0.09 (0.02)	0.27 (0.01)
			P_HD_	0.05 (0.01)	0.79 (0.01)	0.07 (0.01)	0.13 (0.00)
			P_HDC_	0.20 (0.02)	0.76 (0.01)	0.27 (0.03)	0.41 (0.01)
	RDC	JER	C_c_	0.85 (0.01)	0.79 (0.01)	—	—
			P_SEQ1kb_	0.33 (0.03)	0.77 (0.01)	0.39 (0.04)	0.42 (0.01)
			P_SEQ10kb_	0.19 (0.03)	0.75 (0.01)	0.22 (0.03)	0.27 (0.01)
			P_SEQ25kb_	0.07 (0.01)	0.71 (0.01)	0.08 (0.02)	0.17 (0.01)
			P_50K_	0.08 (0.02)	0.76 (0.01)	0.09 (0.02)	0.07 (0.00)
			P_50KC_	0.11 (0.02)	0.60 (0.01)	0.13 (0.02)	0.22 (0.01)
			P_HD_	0.10 (0.02)	0.78 (0.01)	0.11 (0.02)	0.07 (0.00)
			P_HDC_	0.25 (0.03)	0.66 (0.01)	0.29 (0.03)	0.38 (0.01)

SE of reliability, estimated heritability, RF_O_ and RF_P_ are given in parentheses. ncaus, the number of causative mutations; train, training population; val, validation population; set, markers used for prediction; RF_O_, observed reliability factor; RF_P_, predicted reliability factor; JER, Jersey; RDC, Danish Red; C_c_, causative mutations; P_SEQ1/10/25kb_, sequence interval on 1 kb/10 kb/25 kb of the causative mutations; P_50K_ and P_HD_, all markers on the 50K and HD SNP chip, respectively; P_50KC_ and P_HDC_, the closest causative mutations to each 50K and HD marker, respectively.

### Heritabilities for different sets of markers

Estimated heritabilities were very close to the true heritability when prediction was based on the causative mutations. Averaged across scenarios with different number of causative mutations and breeds, the heritability was estimated to be 0.79. Using different sets of markers, the highest heritabilities were obtained with P_50K_ and P_HD_. Here, heritabilities were 0.77 averaged across scenarios with different numbers of causative mutations and 0.78 averaged over breeds. When sequence intervals near the causative mutations were used for prediction, heritabilities decreased when the distance between causative mutations and prediction intervals (*d*) increased. Heritabilities ranged from 0.61 for prediction of JER from RDC for scenario C_50_P_SEQ25kb_ to 0.81 for prediction of RDC from JER for scenario C_250_P_SEQ1kb_. The lowest heritabilities were obtained for P50_KC_ and P_HDC_, with 0.56 averaged across scenarios with different numbers of causative mutations, and 0.64 when averaged over breeds.

### Observed and predicted loss in reliability

**Figure 3:**
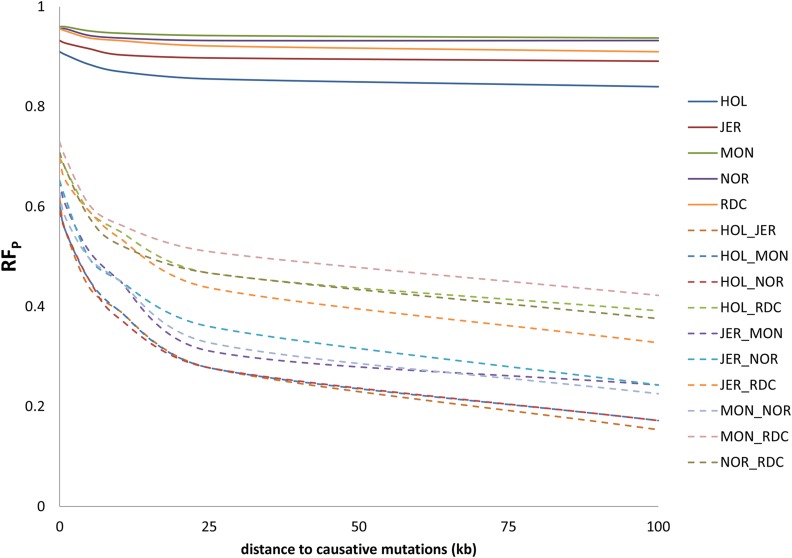
Reliability factor (RF_P_) as a function of the distance between causative mutations and intervals in prediction scenarios for within- and across-breed prediction. HOL, Holstein; JER, Jersey; MON, Montbéliarde; NOR, Normande; RDC, Danish Red.

Averaged across sets of markers and breeds, the difference between observed and predicted RF equaled 0.06, 0.06, and 0.09 for the scenarios with 50, 100, and 250 causative mutations, respectively. Figure 3 show the reliability, RF_O_ and RF_P_ as a function of the number of causative mutations. While RF_P_ increased when the number of causative mutations increased, the reliability and PF_O_ decreased. Averaged across scenarios and breeds, RF_P_ equaled 0.19, 0.21, and 0.26 for C_50_, C_100_, and C_250_, respectively, compared to an RF_O_ of 0.23, 0.23, and 0.17. Consequently, the loss in reliability was overestimated with 50 and 100 causative mutations, but underestimated with 250 causative mutations. With 50 causative mutations, the predicted RF minus the observed RF (RF_P-O_) ranged from –0.13 with P_HD_ to 0.03 with P_50KC_ for prediction of JER, and from –0.07 with P_HD_ to 0.09 with P_50KC_ for RDC. In the scenarios with 100 causative mutations, RF_P-O_ ranged from –0.12 for PHD to 0.04 to P_50KC_ in JER, and from –0.07 to 0.11 for RDC. With 250 causative mutations, RF was underestimated only with P_50K_ and P_HD_, and prediction of JER. RF_P-O_ ranged from –0.04 with P_50K_ to 0.09 with P_SEQ25kb_ for JER, and from 0.06 for P_HD_ to 0.19 for P_50KC_ for RDC.

### Influence of the distance to causative mutations for within- and across-breed prediction

Figure 3 shows RF_P_ for the C_250_P_SEQd_ scenarios as a function of *d* for within- and across-breed prediction. While both within and across breeds, RF_P_ decreases when *d* increases, this decrease was larger across than within breeds. For example, within HOL, RF_P_ decreased from 0.91 to 0.86 when *d* increased from 1 base to 25 kb, while for across-breed prediction in HOL and MON, RF_P_ decreased from 0.60 to 0.28 with the same increase in *d*. Within-breed, RF_P_ was highest for MON and lowest for HOL, and, across breeds, RF_P_ was highest for breed combinations with RDC. The sharpest decrease in RF_P_ was between 1 base and 25 kb. For example, between JER and MON, RF_P_ dropped from 0.65 to 0.28 when *d* increased from 1 base to 25 kb, and further decreased to 0.10 when *d* was 100 kb. All breeds and across-breed combinations showed the same pattern, and more or less a parallel decrease in RF_P_.

### MAF

**Figure 4:**
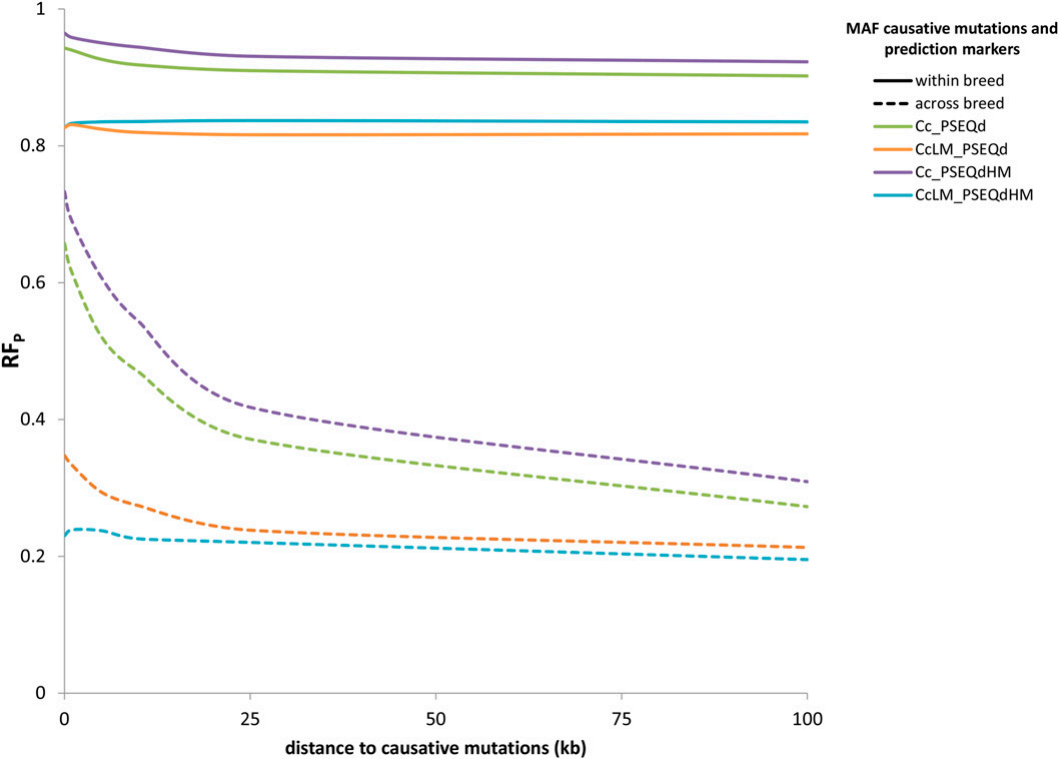
The influence of the MAF of causative mutations and markers on the reliability factor (RF_P_). Cc_PSEQd, no restriction on MAF for either causative mutations or markers; CcLM_PSEQd, MAF causative mutations ≤ 0.10 (no restriction for markers); Cc_PSEQdHM, no restriction for causative mutations, MAF markers > 0.10; CcLM_PSEQdHM, MAF causative mutations ≤ 0.10, MAF markers > 0.10.

The relation between RF_P_ and MAF of causative mutations and markers is shown in Figure 4. RF was lower for causative mutations with a low MAF than for scenarios with both low and high MAF causative mutations. When causative mutations contained both low and high MAF variants, prediction was always slightly better when prediction was based only on high MAF markers, while in the scenarios with only low MAF causative mutations, RF_P_ was generally higher when both low and MAF markers were used. The difference was larger with markers within 25 kb of the causative mutations.

## Discussion

We quantified the loss in prediction reliability due to the use of markers in imperfect LD with causative mutations for different simulation scenarios, both within and across breeds. For some scenarios, we estimated prediction reliabilities, while for others, we used a formula to approximate the loss in prediction reliability. The loss in reliability quickly increased with the distance between markers and causative mutations, especially across breeds, and was influenced by the MAF of both causative mutations and markers.

### Distance between causative mutations and markers

All sets of markers resulted in large decreases in prediction reliabilities compared to prediction using only the causative mutations. This is in line with results from a simulation study by Pérez-Enciso *et al.* (2015), where prediction accuracies decreased rapidly when variants in lower LD with the causative mutations were included. Using only causative mutations for genomic prediction is, however, not realistic, since only a few causative mutations have been identified (Grisart *et al.* 2004; Braunschweig 2010). Alternatively, numerous quantitative trait loci (QTL) have been identified for various traits (Khatkar *et al.* 2004; Daetwyler *et al.* 2008; Cole *et al.* 2011; Höglund *et al.* 2014; Sahana *et al.* 2014), and can be used for genomic prediction. Therefore, we tested scenarios that used sequence intervals near the causative mutations, and increased the distance between the intervals and the causative mutations. While using these sequence intervals resulted in substantial decreases in reliability compared to prediction using the causative mutations, these scenarios did result in higher reliabilities than those obtained using all markers from SNP chips. The reliability rapidly decreased when the distance to causative mutations increased, showing the importance of mapping precision. Several studies using real data have shown that increases in prediction reliability can be obtained using variants associated with QTL (Boichard *et al.* 2012; Brøndum *et al.* 2015; Ober *et al.* 2015; Porto-Neto *et al.* 2015).

### Marker density

The accuracy of genomic prediction is highly dependent on the level of LD between markers and causative variants. Therefore, sequence data can potentially increase prediction accuracy because it contains variants in high LD with the causative mutations. Increasing the marker density adds markers closer to, and in higher LD, with the causative mutations. At the same time, however, markers at a large distance of the causative mutations are added. Therefore, the genomic relationships at markers will be close to the genomic relationships at all loci rather than the genomic relationships at the causative mutations. As a consequence, increasing the density of the SNP markers from 50K to HD did not increase the reliability. Reliability increased only when the markers further away from the causative mutations were discarded, because genomic relationships at markers then approach the genomic relationships at causative mutations. This is in agreement with Erbe *et al.* (2012), who found no difference in prediction accuracy when the marker density was increased from 50K to HD using the GBLUP model. However, when Bayesian variable selection models were used, both Erbe *et al.* (2012) and Hozé *et al.* (2014) found a small increase in prediction accuracy for HD markers compared to 50K markers. In a study using simulated sequence data, MacLeod *et al.* (2014) found increases in prediction accuracies for sequence data compared to HD data when Bayes R was used. GBLUP (VanRaden 2008) uses all markers to construct a genomic relationship matrix, and, therefore, using all markers from the 50K or HD chips will result in a genomic relationship matrix close to the genomic relationship matrix at all loci, while the Bayesian variable selection models (Habier *et al.* 2011) assume the majority of markers have no effect. By including only markers in high LD with causative mutations in the prediction models, Bayesian models result in covariance structures of phenotypes that are closer to those at causative mutations than GBLUP.

### Prediction of RF

For some scenarios, we compared the RF estimated by cross-validation with the RF predicted by the formula of de los Campos *et al.* (2013). While the differences between RF_P_ and RF_O_ are in the same range as those observed by de los Campos *et al.* (2013), both RF_P_ and RF_O_ were influenced by the number of causative mutations, but in opposite direction. When the number of causative mutations increased, RF_P_ increased, while RF_O_ decreased. Consequently, RF_P_ overestimated the loss in prediction reliabilities in the scenarios with 50 and 100 causative mutations, while it is supposed to be a measure of the minimum reduction in prediction reliability. Therefore, with the SEQ data set, we focused on the scenarios with 250 causative mutations, where RF_P_ could be used as an approximation for the minimum loss in reliability. RF_P_ is based on the genomic relationships at causative mutations and markers. When the number of causative mutations increases, genomic relationships at causative mutations become closer to the genomic relationships at all loci, and therefore, closer to any set of randomly selected markers, resulting in a higher RF_P_. On the other hand, in scenarios with fewer causative mutations, prediction reliabilities may be higher because more information is available to accurately estimate their effect. Originally, RF_P_ was developed with reference to a homogeneous population (de los Campos *et al.* 2013), and applying it to across-breed prediction may have resulted in larger deviations from RF_O_ than it would have had for within-breed prediction. RF_P_ compared genomic relationships matrices at markers and causative mutations, and therefore, the way the genomic relationship matrix is constructed is likely to influence RF_P_. In the across-breed scenarios, we used the allele frequencies of both populations to center the genotypes. Alternatively, we could have centered the genotypes based on the allele frequency of each population, which would have resulted in different genomic relationships, which would in turn have influenced RF_P_.

### MAF

The SNPs on the 50K and HD chips are selected to have a higher MAF than most sequence variants (Matukumalli *et al.* 2009). As a consequence, their predictive ability will be better for common variants than for rare variants. If causative mutations are mainly variants with low MAF, using sequence data for the prediction of causative mutations could increase prediction accuracy. Druet *et al.* (2014) found that using sequence data increases prediction accuracy particularly for rare causative mutations. In our results, including low MAF variants in the prediction intervals increased RF_P_ only if all causative mutations had low MAF and the variants were very close to the causative mutations. When the distance between intervals and causative mutations increased, including low MAF variants still resulted in a slightly higher RF_P_ across breeds, while within-breed RF was higher when low MAF variants were excluded. When causative mutations included both low and high MAF variants, excluding low MAF variants always resulted in a higher RF_P_. The LD between a rare variant and a causative mutation will generally be high if only the causative variant is rare and the distance between both variants is very small. Therefore, the advantage of using rare variants for genomic prediction might be limited, especially when GBLUP is used. Furthermore, for across-breed prediction, the number of rare variants shared between breeds is expected to be low, due to their low frequency, their increased fixation risk due to drift, and their generally younger age compared to high MAF mutations.

### Across-breed genomic prediction

LD within breeds is larger and conserved over longer distances than across breeds (de Roos *et al.* 2008). As a consequence, RF_P_ was lower and decreased faster with an increasing distance across breeds than within breeds. Differences in LD within breeds, and in the amount of variants shared and the LD preserved across breeds, can explain differences between the different breeds and different breed combinations. HOL used for the analysis consisted of different Holstein populations, originating from several countries, while for the other breeds sequences originated from one country. This most likely explains why RF_P_ for HOL was lower than for the other breeds.

### Limitations and further studies

In our study, we only focused on the influence of LD between markers and causative mutations on the prediction of genomic relationships at causative mutations. In practice, there are many other factors influencing the accuracy of genomic prediction that we did not take into account. Across-breed prediction requires, besides high LD between causative mutations and markers, causative mutations to be segregating across breeds, and to have similar allele substitution effects in different breeds. In the across-breed scenarios, causative mutations were sampled from all causative mutations segregating in at least one of the breeds. In practice, the proportion of QTL segregating across breeds may be smaller than those presented in Table 2 (Raven *et al.* 2014). Furthermore, by predicting genomic relationships rather than breeding values, we did not consider scenarios where allele substitution effects vary across breeds, due to, *e.g.*, dominance and epistasis. Our results suggest that inclusion of sequence variants near causative mutations can increase across-breed prediction. Because some of the assumptions made in the simulation of the phenotypes are likely to be violated in real populations, our results should be interpreted as maximum gains that could be obtained by using variants in close LD with the causative mutations. In practice, gains would be expected to be smaller, due to factors other than LD limiting across-breed prediction. Further studies are required to investigate the effect of the proportion of causative mutations segregating across breeds, and differences and genetic heterogeneity on the accuracy, and advantage, of sequence variants for across-breed prediction.

### Conclusions

Our results show that sequence data can potentially improve genomic prediction. While using the full sequence directly for prediction is not likely to improve prediction, using only variants close to the causative mutations could substantially increase reliability. This was especially the case for across-breed prediction, where reliabilities dropped quickly when the distance between causative mutations and markers increased. Using rare sequence variants improved prediction only when they were very close to rare causative mutations. To exploit the potential advantages of sequence data for genomic prediction, careful selection of markers is essential.

## Supplementary Material

Supplemental Material
