# Supplementary material for: Using Sequence Variants in Linkage Disequilibrium with Causative Mutations to Improve Across-Breed Prediction in Dairy Cattle: A Simulation Study
Source: G3 (Bethesda). 2016 Jun 13;6(8):2553–61. doi: 10.1534/g3.116.027730 (PMC4978908; doi:10.1534/g3.116.027730)
Supplement: Supplemental Material [file supp_6_8_2553__index.html]

Using Sequence Variants in Linkage Disequilibrium with Causative Mutations to Improve Across-Breed Prediction in Dairy Cattle: A Simulation Study — Supplemental Material 

# Using Sequence Variants in Linkage Disequilibrium with Causative Mutations to Improve Across-Breed Prediction in Dairy Cattle: A Simulation Study

## Supplemental Material for van den Berg *et al.*, 2016

**Files in this Data Supplement:**

- File S1 - SEQ dataset. (.zip, 56 MB)
- File S2 - IMP dataset. (.zip, 111 MB)
